# Supplementary material for: The architecture of assisted colonisation in sea turtles: building new populations in a biodiversity crisis
Source: Nat Commun. 2022 Mar 24;13:1580. doi: 10.1038/s41467-022-29232-5 (PMC8948361; doi:10.1038/s41467-022-29232-5)
Supplement: Supplementary file 1 — Supplementary Information [file 41467_2022_29232_MOESM1_ESM.pdf]

Supplementary information

Table S1. Pairwise F_{ST} values between Grand Cayman (n=115) and Little Cayman (n=34) hatchlings and the Cayman Turtle Farm (CTF, n=257) using mitochondrial markers (above diagonal) and microsatellite markers (below diagonal). Significant values are in bold (p-value ≤ 0.05). Only one inferred nest per female was considered to avoid pseudo-replication (n=406).

	Grand Cayman	Little Cayman	CTF
Grand Cayman	0.000	0.026	-0.004
Little Cayman	**0.011**	0.000	**0.036**
CTF	**0.004**	**0.016**	0.000

Table S2. Minimum genetic census of Grand Cayman and Little Cayman Islands with related sex ratio as provided by parentage analyses and significance of the deviation from a 1:1 ratio as computed with the Chi-squared test.

	**Males**	**Females**	**Sex Ratio**	**Chi-squared**	**p-value**
**Little Cayman**	28	32	0.875	0.333	0.855
**Grand Cayman**	99	100	0.990	0	1

Table S3. Details of the six Linear Mixed-Effects Models performed on the nests that were assigned to wild sampled females according to parentage analysis (n = 149). For each model we detail the Response variable and the Random and Fixed effects considered, as well as the intercept. F1-F5 models assess effects on fitness, and H1 model assesses effects on hatchling heterozygosity. For each tested fixed effect and the intercept, we detail the χ^2 value, the degrees of freedom (d.f.), and the p-value. For each model, we indicate the R^2 for the full model (Model R^2) and the fixed effects (F.E. R^2), as well as the error structure used. Bold numbers indicate significant two-sided tests after FDR correction for multiple comparisons.

Model	Response	Random Effects	Fixed Effects	χ^2	d.f.	p-value	F.E. R^2	Model R^2	Error Structure
F1	Viability	Mother ID	Mother Heterozygosity	0.028	1	0.867	0.239	0.473	Gaussian
		Year	Hatchling Heterozygosity	1.012	1	0.314			
			Fertilization success	29.259	1	**0.000**			
			Curved Carapace Length	2.938	1	0.087			
			Intercept	*2.045*	*1*	*0.153*			
F2	Viability	Mother ID	Mother – Farm relatedness	0.308	2	0.857	0.062	0.217	Gaussian
		Year	Nest – Farm relatedness	1.247	1	0.264			
			Lay date (Quartile)	4.436	3	0.218			
			Intercept	*0.044*	*1*	*0.834*			
F3	Fertilization success	Mother ID	Mother Heterozygosity	1.469	1	0.226	0.111	0.158	Gaussian
		Year	Hatchling Heterozygosity	0.000	1	0.997			
			Curved Carapace Length	1.252	1	0.263			
			Clutch Size	8.550	1	**0.003**			
			Intercept	*1.011*	*1*	*0.315*			
F4	Fertilization success	Mother ID	Mother – Farm relatedness	0.782	2	0.676	0.087	0.285	Gaussian
		Year	Nest – Farm relatedness	0.997	1	0.318			
			Lay date (Quartile)	5.971	3	0.113			
			Intercept	*0.002*	*1*	*0.966*			
F5	Clutch size	Mother ID	Mother Heterozygosity	0.115	1	0.735	0.215	0.328	Gaussian

		Year	Mother – Farm relatedness	0.548	2	0.760			
			Lay date (Quartile)	0.868	3	0.833			
			Curved Carapace Length	14.399	1	**0.000**			
			Intercept	*13.941*	*1*	*0.000*			
H1	Hatchling Heterozygosity	Mother ID	Mother – Farm relatedness	0.351	2	0.839	0.044	0.270	Gaussian
		Year	Nest – Farm relatedness	0.928	1	0.087			
			Mother Heterozygosity	0.382	1	0.536			
			Mother – Farm rel. * Mother Heterozygosity	0.351	1	0.553			
			Intercept	*18.612*	*1*	*0.000*			

Table S4. Female-nest pairs recorded during night patrols while nesting. For these nests no hatchling was sampled or genotyped. Some females laid more than one nest and therefore their ID is repeated. na: no geographic coordinates were recorded for these nests.

Year	Female ID	Nest ID	Latitude	Longitude
2013	G001	80	19.366360	-81.396160
2013	G001	112	19.366850	-81.396910
2013	G002	81	19.366700	-81.396600
2013	G002	116	na	na
2013	G003	85	19.364080	-81.393240
2013	G003	117	19.367140	-81.397350
2013	G005	125	19.366440	-81.396270
2013	G005	327	19.363430	-81.392370
2013	G006	115	19.369480	-81.402030
2013	G008	165	19.356590	-81.387120
2013	G008	242	19.355920	-81.386870
2013	G014	241	19.386630	-81.411200
2013	G016	267	19.354780	-81.386420
2013	G020	329	19.363590	-81.392600
2013	G022	396	19.386270	-81.412280
2014	G027	296	na	na

Table S5. Detailed results of the post-hoc Wilcoxon rank sum tests comparing the observed heterozygosity values among the CTF breeding stock subgroups, wild females and nests related (RH) or unrelated (UH) to the CTF (n=634 individuals), as shown in Figure 5b. The values of the W statistic are shown above the diagonal while the p-values of the tests are shown below the diagonal. Significant values are in bold (p-value ≤ 0.05).

	Founder	$F1_{(mixed)}$	$F1_{(1995)}$	Wild	RH	UH
Founder	-	335.5	1881.5	671.0	3294.5	362.5
$F1_{(mixed)}$	**0.008**	-	4633.5	694.0	7921.5	898.5
$F1_{(1995)}$	0.093	0.144	-	4019.0	30488.0	3410.0
Wild	0.674	**0.000**	**0.003**	-	7062.5	741.0
RH	0.583	**0.001**	**0.007**	0.143	-	4322.5
UH	0.112	0.437	0.620	**0.008**	**0.050**	-

Table S6. List of mitochondrial DNA D-loop haplotypes found in the analysed samples (Supplementary Data 1), including their accession number.

D-loop haplotype	Genbank Accession number
**CM-A1.1**	JF308465
**CM-A1.2**	JF308466
**CM-A3.1**	JN632497
**CM-A3.4**	OM743492
**CM-A5.1**	JN632498
**CM-A13.1**	JX306007
**CM-A17.1**	JQ420802
**CM-A18.2**	JX306008
**CM-A22.1**	KT581619
**CM-A27.1**	MH025956
**CM-A34.1**	OM743493
**CM-A80.1**	OM743494
