# Supplementary material for: The architecture of assisted colonisation in sea turtles: building new populations in a biodiversity crisis
Source: Nat Commun. 2022 Mar 24;13:1580. doi: 10.1038/s41467-022-29232-5 (PMC8948361; doi:10.1038/s41467-022-29232-5)
Supplement: Supplementary file 3 — Description of Additional Supplementary Information [file 41467_2022_29232_MOESM3_ESM.pdf]

Description of Additional Supplementary Files

Title: Supplementary Data 1
Description: Information of each genotyped hatchling at the 13 microsatellite loci (A6-Or7). Hatchling ID identifies the nest and year (ǂ symbol denotes nests whose female has been seen laying the eggs). Inferred Mother and Father ID according to genetic analyses with COLONY is provided. CTF genetic relatedness of each hatchling. For hatclings assigned to a genotyped wild mother, geographic and fitness information of the nest is given when available. Fo each of these nests we provide geographic coordinates and estimated lay date, as well as number of eggs (Clutch size), frequency of eggs that hatched or developed an embryo (Fertilization success), and frequency of hatched individuals from eggs with developed embryos (Viability).

Title: Supplementary Data 2

Description: Clutch relatedness to CTF females by sibship analyses. The table shows for each clutch the number of captive females related by ML-Relate and/or Coancestry. For ML-Relate we display the number of females found in each of the relatedness categories (i.e. PO = parent-offspring, FS = full sibling, HS = half sibling). For Coancestry the table shows the number of females found with lower bound of 95% confidence interval higher than 0.0001 and its r value higher than or equal to 0.3070.
