# Supplementary material for: The architecture of assisted colonisation in sea turtles: building new populations in a biodiversity crisis
Source: Nat Commun. 2022 Mar 24;13:1580. doi: 10.1038/s41467-022-29232-5 (PMC8948361; doi:10.1038/s41467-022-29232-5)
Supplement: Supplementary file 6 — Reporting Summary [file 41467_2022_29232_MOESM6_ESM.pdf]

nature research

Corresponding author(s): Carlos Carreras
Last updated by author(s): Jul 22, 2021

Reporting Summary

Nature Research wishes to improve the reproducibility of the work that we publish. This form provides structure for consistency and transparency in reporting. For further information on Nature Research policies, see our Editorial Policies and the Editorial Policy Checklist.

Statistics

For all statistical analyses, confirm that the following items are present in the figure legend, table legend, main text, or Methods section.

n/a	Confirmed	
☐	☒	The exact sample size (*n*) for each experimental group/condition, given as a discrete number and unit of measurement
☒	☐	A statement on whether measurements were taken from distinct samples or whether the same sample was measured repeatedly
☐	☒	The statistical test(s) used AND whether they are one- or two-sided<br>*Only common tests should be described solely by name; describe more complex techniques in the Methods section.*
☐	☒	A description of all covariates tested
☐	☒	A description of any assumptions or corrections, such as tests of normality and adjustment for multiple comparisons
☐	☒	A full description of the statistical parameters including central tendency (e.g. means) or other basic estimates (e.g. regression coefficient) AND variation (e.g. standard deviation) or associated estimates of uncertainty (e.g. confidence intervals)
☐	☒	For null hypothesis testing, the test statistic (e.g. F, t, r) with confidence intervals, effect sizes, degrees of freedom and P value noted<br>*Give P values as exact values whenever suitable.*
☒	☐	For Bayesian analysis, information on the choice of priors and Markov chain Monte Carlo settings
☒	☐	For hierarchical and complex designs, identification of the appropriate level for tests and full reporting of outcomes
☐	☒	Estimates of effect sizes (e.g. Cohen's *d*, Pearson's *r*), indicating how they were calculated

Our web collection on statistics for biologists contains articles on many of the points above.

Software and code

Policy information about availability of computer code

Data collection	No software was used for data collection
Data analysis	All the software used for data analysis, including the version used, is mentioned in the manuscript with the appropriate citation.

For manuscripts utilizing custom algorithms or software that are central to the research but not yet described in published literature, software must be made available to editors and reviewers. We strongly encourage code deposition in a community repository (e.g. GitHub). See the Nature Research guidelines for submitting code & software for further information.

Data

Policy information about availability of data

All manuscripts must include a data availability statement. This statement should provide the following information, where applicable:

- Accession codes, unique identifiers, or web links for publicly available datasets
- A list of figures that have associated raw data
- A description of any restrictions on data availability

All genotypes, parentage analyses detailed results and field data collected are included in the Supplementary Information files. Sequences and Genebank Accession Numbers of the d-loop haplotypes can be found in the marine turtle mtDNA haplotypes open access database maintained by the 'Archie Carr centre for sea turtle research' of the university of Florida (https://accstr.ufl.edu/resources/mtdna-sequences/)

Field-specific reporting

Please select the one below that is the best fit for your research. If you are not sure, read the appropriate sections before making your selection.

☐ Life sciences ☐ Behavioural & social sciences ☒ Ecological, evolutionary & environmental sciences

For a reference copy of the document with all sections, see nature.com/documents/nr-reporting-summary-flat.pdf

Ecological, evolutionary & environmental sciences study design

All studies must disclose on these points even when the disclosure is negative.

Study description	In this study we ran population genetic analysis of green turtles. We used microsatellite markers and mitochondrial markers to genotype our samples. The results of the genetic analysis were used to run parentage and sibship analysis, estimate population differentiation using pairwise Fst, and estimate the census size. We combined the genetic data with ecologial data to understand the degree of nest-site fidelity of the studied individuals and to run Linear Mixed Effect Models.
Research sample	Our sample comprised 320 hatchlings from the Cayman Islands. We had samples for 3 nesting seasons for Grand Cayman and 2 nesting season for Little Cayman Island. We also considered genotypes of 57 wild females and 257 captive females from the Cayman Turtle Center published in a previous study. Based on the existing bibliography, this sample size is considered adequate for the type of analysis run in this study for population genetic analysis of sea turtles.
Sampling strategy	Sampling is regularly performed by the Department of Environment (DOE) of the Cayman Islands Government to monitor nesting beaches of marine turtles on the islands of Little Cayman and Grand Cayman, in the Caribbean. Samples were taken from the hatchling's margin of the carapace and up to three hatchlings per nest were sampled (only one per nest genotyped). Samples were obtained with a scalpel blade and stored in 100% ethanol.
Data collection	Data collection is regularly performed by the Department of Environment (DOE) of the Cayman Islands Government to monitor nesting beaches of marine turtles on the islands of Little Cayman and Grand Cayman, in the Caribbean. From the DOE database we gathered information regarding nesting date, GPS location as well as the following reproductive parameters (Supplementary Information): number of eggs, number of eggs which developed an embryo, and viability (i.e. number of eggs hatched from those with developed embryos), following standard procedures.
Timing and spatial scale	Data and samples were collected between April and October, which corresponds to green turtle nesting season in the Caribbean. In Grand Cayman Islands, the collection was performed in 2013, 2014 and 2015. In Little Cayman Island the collection was performed in 2014 and 2015.
Data exclusions	No data were excluded from the analysis.
Reproducibility	This study does not include experimental steps.
Randomization	This study does not include randomization.
Blinding	Blinding was not relevant for this study as no experimental bias is expected.

Did the study involve field work? ☒ Yes ☐ No

Field work, collection and transport

Field conditions	Samples were collected on sea turtle nesting beaches in Grand Cayman and Little Cayman, Cayman Islands.
Location	Grand Cayman (19.3222ºN, 81.2409ºW) and little Cayman (19.6897ºN, 80.0367ºW)
Access & import/export	Samples were collected by the Cayman Islands Department of Environment (Cayman Islands Government), in accordance with national conservation legislation. and exported under CITES permit (export #2015/KY/000807 and UK import #540953/01).
Disturbance	Sampling was minimally invasive and followed stardard protocols (e.g. Wright et al. (2012). Mol. Ecol. 21, 3625–3635.)

Reporting for specific materials, systems and methods

We require information from authors about some types of materials, experimental systems and methods used in many studies. Here, indicate whether each material, system or method listed is relevant to your study. If you are not sure if a list item applies to your research, read the appropriate section before selecting a response.

Materials & experimental systems

n/a	Involved in the study	
☒	☐	Antibodies
☒	☐	Eukaryotic cell lines
☒	☐	Palaeontology and archaeology
☐	☒	Animals and other organisms
☒	☐	Human research participants
☒	☐	Clinical data
☒	☐	Dual use research of concern

Methods

n/a	Involved in the study	
☒	☐	ChIP-seq
☒	☐	Flow cytometry
☒	☐	MRI-based neuroimaging

Animals and other organisms

Policy information about studies involving animals; ARRIVE guidelines recommended for reporting animal research

Laboratory animals	No laboratory animals were used in this study
Wild animals	Minimmally-invasive samples were collected from the carapace margin of live, non-emergent green turtle hatchlings (Chelonia mydas), from nests in Grand Cayman and little Cayman, Cayman Islands. Hatchlings were released at the capture location after de collection of samples
Field-collected samples	Tissue samples were stored in 100% ethanol at 4 degrees Celsius or -20 degrees Celsius.
Ethics oversight	Field collection of samples was conducted and overseen by the Cayman Islands Department of Environment, as an authorised agent of the Cayman Islands Government.

Note that full information on the approval of the study protocol must also be provided in the manuscript.
